# PDT-Induced Activation Enhanced by Hormone Response to Treatment

**DOI:** 10.3390/ijms241813917

**Published:** 2023-09-10

**Authors:** Wojciech Domka, Dorota Bartusik-Aebisher, Maria Przygoda, Klaudia Dynarowicz, Jerzy Tomik, David Aebisher

**Affiliations:** 1 Department of Otolaryngology, Medical College of the University of Rzeszów, 35-959 Rzeszów, Poland; w.domka@gazeta.pl; 2 Department of Biochemistry and General Chemistry, Medical College of the University of Rzeszów, 35-959 Rzeszów, Poland; dbartusikaebisher@ur.edu.pl; 3 Students English Division Science Club, Medical College of the University of Rzeszów, 35-959 Rzeszów, Poland; maria.przygoda@interia.pl; 4 Center for Innovative Research in Medical and Natural Sciences, Medical College of the University of Rzeszów, 35-310 Rzeszów, Poland; kdynarowicz@ur.edu.pl; 5 Department of Otolaryngology, Collegium Medicum, Jagiellonian University, 30-688 Krakow, Poland; j.tomik@uj.edu.pl; 6 Department of Photomedicine and Physical Chemistry, Medical College of the University of Rzeszów, 35-959 Rzeszów, Poland

**Keywords:** photodynamic therapy, PDT, tumor, acute-phase response, glucocorticoids

## Abstract

Photodynamic therapy (PDT) is a medical treatment with the use of a photosensitizing agent (PS), which, when activated by light, results in selective tissue damage with a cytotoxic effect on tumor cells. PDT leads to the induction of an acute-phase response, which results in the involvement of adrenal glucocorticoid (GC) hormones. PDT, by activating the hormonal response, affects the treatment of cancer. GC release is observed due to adrenal activity, which is driven by changes in the hypothalamic pituitary–adrenal axis triggered by stress signals emanating from the PDT treated tumor. The hormones released in this process in the context of the PDT-induced acute-phase response perform many important functions during anticancer therapy. They lead, among other things, to the systemic mobilization of neutrophils and the production of acute-phase reagents, and also control the production of immunoregulatory proteins and proteins that modulate inflammation. GCs can radically affect the activity of various inflammatory and immune cells, including the apoptosis of cancer cells. A better understanding of the modulation of GC activity could improve the outcomes of cancer patients treated with PDT.

## 1. Introduction

Despite the continuous development of science towards an understanding of the mechanisms of cancer formation, there were large clinical trials conducted in recent years [1,2], which have resulted in the creation of a new generation of targeted drugs [3]. However, the treatment of some cancers is still ineffective. These facts have led to a further search for solutions placing the emphasis on other therapeutic approaches, which include, among others, photodynamic therapy (PDT). Despite its low popularity, this is a well-tested, clinically approved, and effective method of cancer treatment [4,5,6]. Accurate knowledge of PDT mechanisms gives a chance for better understanding of this treatment [7]. Additionally, PDT causes cellular acting, including an impact on the endocrine and immunological pathways. With the results of experimental and clinical research, it is possible to create a more precise anticancer therapy using targeted action on a specific hormonal and/or immunological mechanism for the treatment of patients with cancer. PDT’s results can bring better clinical effects and the lowest possible risk of complications and side effects.

### 1.1. Photodynamic Therapy-Mechanism

**Figure 1:**
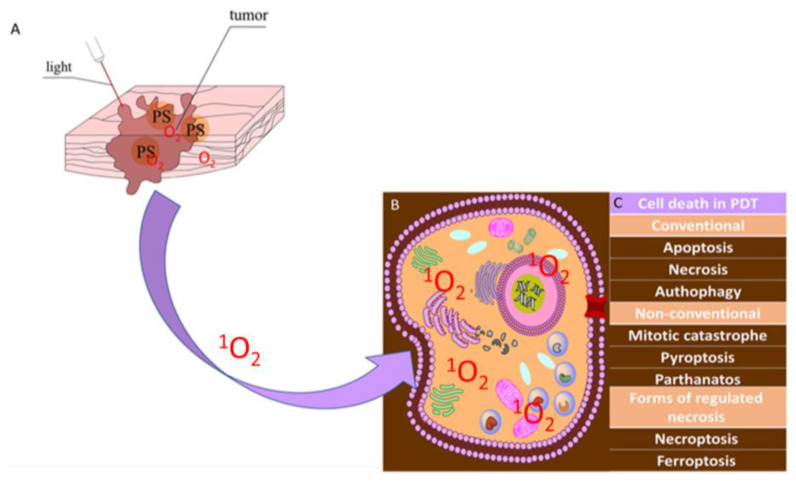
Principles of PDT. (**A**) The PS selectively accumulates in tumor cells after local or systemic administration. Light of the appropriate wavelength leads to activation of the PS, and, in the presence of ^3^O_2_, induces a photochemical process, culminating in the production of ^1^O_2_. (**B**) Singlet oxygen located in cells, (**C**) categories of cell death after PDT.

The action of PDT on cancer cells is based on three mechanisms: damage to tumor vessels, induction of a strong inflammatory reaction, and a direct cytotoxic effect on cancer cells [8]. These three mechanisms are interrelated, and their quantitative contributions depend, among other things, on the dose and type of PS, oxygen concentration in the cancer cells, the wavelength of light, the total dose of PS, irradiation time, and fluctuation rate, meaning the time between exposure to light and administration of PS [9]. As shown in Figure 1, PDT is a two-stage procedure consisting of PS administration and successive irradiation of the tumor [10,11,12,13,14,15,16]. The use of PDT is possible in the case of a neoplastic tumor of any organ with the use of flexible optical-fiber devices for precise irradiation of the treated tissues. In current analyses of cell death, the cell pathways paraptosis, parthanatos, mitotic catastrophe, pyroptosis, necroptosis, and ferroptosis have been demonstrated, in addition to the traditional division into apoptosis, autophagy-related cell death, and necrosis [17].

The selectivity of PDT depends on the precise delivery of the light beam and on the affinity and localization capacity of the PS in neoplastic lesions [18,19,20].

PDT is a method with three components: a PS [21], light with an appropriate wavelength, and oxygen [22,23,24]. When all three elements are combined, singlet oxygen (^1^O_2_) is produced, which is a highly reactive product that has a toxic effect on cells [25].

The destruction of cancer cells under the influence of PDT may occur through apoptosis and necrosis [26,27].

Necrosis is a mechanism of cell death that is recognized by observing the vacuolization of the cytoplasm as well as the breakdown of the cell membrane [28]. The consequence of this process is the transport of mediators and other components of the cytoplasm outside the extracellular space, causing inflammation.

Apoptosis, on the other hand, is a genetically encoded mechanism of programmed cell death that depends on energy [29]. It proceeds with chromatin condensation, splitting of chromosomal DNA into internucleosomal fragments, wrinkling of the membrane without its disintegration, cell shrinkage, and the formation of apoptotic bodies [29,30,31]. Apoptosis is initiated by photodamage to the mitochondria, which releases cytochrome c into the cytosol [32]. During this time, the lysosome releases lysosomal proteases into the cytosol. The consequence of this is the pro-apoptotic cleavage of a protein called Bid into a more active form called tBid. This short form of Bid binds to the mitochondrial membrane, and the apoptosis process is initiated [33].

ROS formed during PDT are a factor that allows the triggering and generation of autophagosomes [34]. Autophagy causes the death of cancer cells and, as a result, certain reactions that are apoptotic are impaired. In this mechanism, certain reactions cause photodamaged factors to be unsuitable for recycling. [35].

### 1.2. Reactive Oxygen Species

PDT depends largely on the nature and persistence of ROS [36]. They are responsible for the changes taking place in the cancer cells. Chromatin condensation is a result of ROS in cells and tissues for cytoplasm swelling or the elimination of intercellular boundaries [37,38].

The main sources of intracellular ROS are the mitochondria and the endoplasmic reticulum (ER) [39]. Other cellular components, such as enzymes associated with the endoplasmic reticulum, cytoplasmic enzyme systems, and the surface of the cell membrane also contribute to the production of ROS.

Hydroxyl radicals and singlet oxygen both exhibit high reactivity [40].

In the PDT mechanism, absorption of a photon by the PS and the subsequent intersystem crossing to a triplet, excited state of the PS allows for energy transfer by collision with ^3^O_2_ to give ^1^O_2_ [41]. ^1^O_2_ is the most important reactive oxygen species produced in a tissue-based PS. Singlet oxygen is a highly reactive electrophile that is toxic to cells. Delivering a precise dose of singlet oxygen to tumors, affording control, is a current objective in cancer therapy and photomedicine. The light used to excite the PS during the clinical procedure is usually supplied through a catheter containing optical fibers, which serve to generate the excited PS and cytotoxic ^1^O_2_ [42].

ROS are formed by the partial (incomplete) reduction of molecular oxygen in the cell [43]. All ROS types have free electrons in their outermost shell or have unstable bonds [44]. The ROS group includes neutral molecules, ions, and radicals [45]. ROS arise not only as a product of PDT therapy but occur naturally during metabolic reactions and in the process of cellular respiration [46]. Naturally formed ROS have a number of significant functions in the body. The most important ones include antimicrobial activity [47], initiation of the apoptosis process, participation in metabolic reactions, and modification of information transmission pathways to the cell [48]. In recent years, specific ROS molecules in redox signaling pathways have been analyzed and assessed. As a result, the role of the oxidants used in various pathological conditions has been confirmed, mainly in diseases of the immune system, circulatory system, and nervous system; in skeletal muscles and metabolic regulation; in the process of maturation and aging of cells; and in neoplastic mechanisms [49].

ROS have a destructive positive effect (e.g., on neoplastic tissues in the PDT reaction that disrupt the cell structure of pathological tissues) and at the same time have a negative destructive effect in the case of fighting healthy cells and excessively initiating the apoptotic process [50]. ROS contribute to the regulation and maintenance of the internal homeostasis of cells and tissues [51]. In recent years, anticancer therapy based on ROS-mediated cell apoptosis has made significant progress in the universality and availability of this treatment method [52]. The Food and Drug Administration (FDA) has approved PDT as a therapy. The European Medicines Agency has also approved PDT as a therapy. Both institutions treat PDT as an acceptable therapy for solid tumors and as a palliative therapy. As a result of PDT, traumatic cellular changes occur, and oxidative stress is triggered in the tumor tissue through an acute inflammatory process. The death of immunogenic cells leads to the release of molecular patterns that activate innate immunity. Such mechanisms lead to the activation of adaptive immunity [53]. The place where PDT occurs reflects the depth of tissue penetration by light. The wavelength of the light sources required for PDT is determined by the absorption spectrum of the PS used in the therapy [50,54]. ^1^O_2_ has lifetimes from microseconds to milliseconds. The diffusion distance of ^1^O_2_ ranges from nanometers to millimeters [55,56,57]. Cancer cells are typical examples in biology where hypoxic processes take place. However, PDT needs ground-state triplet O_2_ for production of ^1^O_2_. Equations (1) and (2) show the standard PDT reaction in cells [56,57,58,59]:

PS + visible light (hν) -> PS*(1)

PS* + ^3^O_2_ -> PS + ^1^O_2_ (2)

PDT usually involves the injection or topical application of a PS, which accumulates in the desired target cells, which are then irradiated by light of a specific wavelength [60]. Apoptosis causes cell death through necrosis accompanied by an acute local inflammatory response that is involved in the removal of dead cells and the restoration of normal tissue homeostasis. Table 1 presents the mechanisms resulting from PDT and the triggering of the immune response in various cell death categories. All cell death modalities for the photodynamic therapy of cancer were discussed in a review by Mishchenko et al. [17].

PDT leads to an immune response at the cellular level. In this way, distant metastases can also be damaged by the same mechanism. The complex relationship between PS, light, light delivery rate, and antigen expression may play a role in stimulating the immune system [69]. PDT results from the induction of the acute-phase response to treatment by activating the hormonal response.

PDT leads to endothelial cell damage. This phenomenon causes the formation of thrombogenic sites in the lumen of the vessel. The consequence of this is the initiation of a physiological cascade of reactions, including platelet aggregation and the release of vasoactive molecules as well as leukocyte adhesion. This also has the effect of increasing vascular permeability and vasoconstriction. It is known that there are three main mechanisms of action of PDT: vascular (occuring by 24 h), cellular, and immune responses [70].

A study published by Huis et al. reported that the acute inflammatory response after PDT occurs within 24 h as a result of damage-associated-molecular-pattern (DAMP) exposure and complement activation promoted by increased vascular adhesion and permeability at the tumor site. The number of neutrophils increases within 24 h, and the number of infiltrating macrophages and mast cells increases within 72 h. Over the next two weeks, lymphocytes accumulate at the site of the tumor. The adaptive response can last for two weeks and is accompanied by high levels of IL-6, TNF-α, and IFN-γ. After two weeks, immune cells are no longer detected in the area treated with PDT [71].

In another study from 2023 by Jin et al., [72] a number of studies have shown that immunotherapy combined with PDT enhances the results of anti-tumor drugs and reduces tumor immune escape. PDT combined with immunotherapy does not induce resistance. This study also confirms that PDT induces acute inflammation and triggers the release of cytokines and stress-response proteins. Firstly, neutrophils are activated in the bloodstream and migrate through blood vessels to infectious or injured sites to kill cancer cells and DAMPs release. Meanwhile, blood vessel injury and tumor cells also attract macrophage infiltration, which regulates macrophage polarization and enhances macrophage phagocytosis of tumor cells. Natural killer cells (NK cells) and dendritic cells (DCs) activate adaptive immune cells, such as monocytes, cytotoxic T lymphocytes (CTLs), and B cells, to enhance the overall immune response by releasing cytokines [72].

However, hypoxia can reduce the efficacy of PDT. Because both tumor growth and PDT need oxygen, its consumption can exacerbate tumor hypoxia. Therefore, to improve this situation, new generations of PSs, such as upconversion nanoparticles, have to be designed [73].

## 2. Results

**Figure 2:**
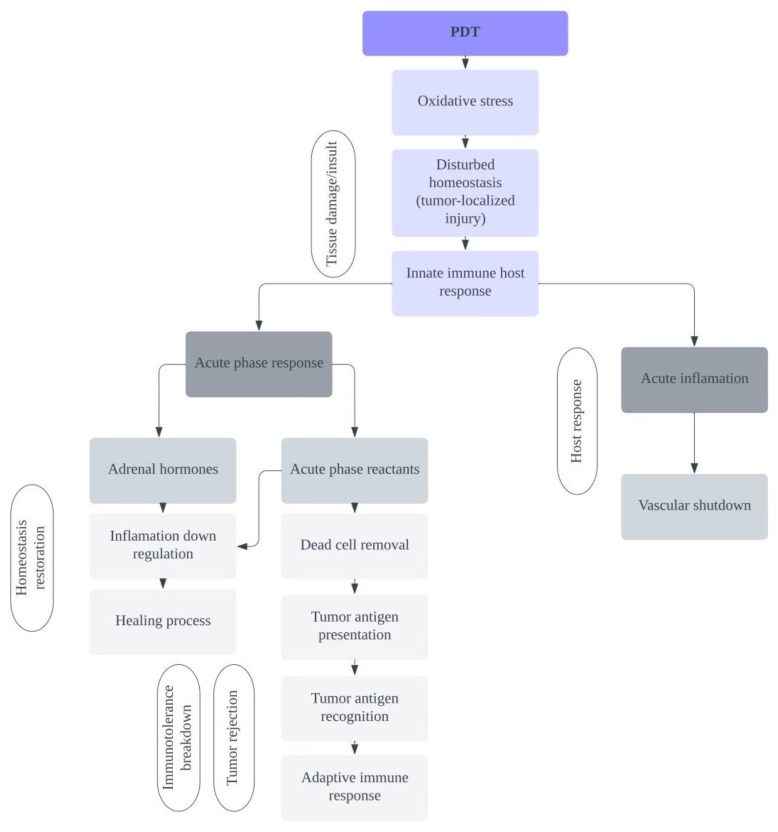
Demonstration of the activation process of the acute-phase response related to PDT cancer cell treatment and schematic representation of the contributions of adrenal hormones and reagents.

The effects of treatment with the use of PDT include disturbed internal balance (homeostasis), which results in the induction of strong defense mechanisms in the host organism and often stimulation of an immune response against the treated tumor [74,75,76]. PDT in the host’s organism aims to use the body’s resources to produce a protective reaction to bring about the restoration of homeostasis. The main process leading to homeostasis is the acute-phase response [77,78,79], which was clearly established by Korbelik et al., who concluded in their studies that tumor PDT induces a strong acute-phase response in hosts [79], which is largely due to inflammatory cytokines, mainly IL-6 [80], as represented in Figure 2.

PDT initiates various forms of destruction of cancer cells. This is so-called immunogenic cell death, which is associated with severe inflammation leading to destruction. ATP releases dendritic cells to the site of the tumor, and cells such as calreticulin contribute to the uptake of tumor antigens by these cells. A strong immune response arises that is dependent on IL-1β and IL-17. This is mediated by IFN-γ activating CD8+ cytotoxic T cells. They migrate through the body, contributing to the destruction of cancer cells, both in the center of the tumor (primary site of irradiation) and in further unexposed neoplastic lesions (metastasis) [81,82]. The immune response induced by PDT stimulates a number of related processes, i.e., the apoptosis and necrosis of cancer cells and inflammatory cells. The stress generated in the endoplasmic reticulum under the influence of PDT combats cancer cells by inducing an active immune subprogram for apoptotic cell death, the prerequisites for which are surface exposure to calcium reticulin, dendritic cell maturation, and activation of T lymphocytes [83].

### 2.1. Involvement of the Hypothalamic-Pituitary-Brain Axis during PDT Treatment

The hallmarks of the acute-phase response in the body are the release of reactants such as proteins, leukocytes, and neutrophils [78] and activation of the hypothalamic-pituitary (HPA) hormone axis.

Stimulation of the HPA axis leads to the release of adrenal corticosteroid hormones. The duration of HPA axis activity is influenced by glucocorticoids. They brake HPA-axis activity at the level of the hypothalamus and pituitary gland. Additionally, the HPA axis is regulated independently of glucocorticoids.

Upon application of PDT, a number of reagents sequestered at the tumor site are released. These acute-phase reagents include complement proteins and pentraxins. Pentraxins specialize in opsonizing dying cells to remove them. The main mediators involved in promoting the PDT-induced acute-phase response of the tumor are glucocorticoid hormones and the cytokine IL-6 [84]. After activation of the HPA axis, release of adrenal glucocorticoids takes place. The inflammatory cytokines produced in PDT action are starting points causing peripheral neural circuits to support the neuronal inflammatory process [85]. Such signals are propagated to the tractus solitarius nucleus in the brainstem via the vagus nerve and then transmitted to the paraventricular nuclei in the hypothalamus, activating the HPA axis [85].

Korbelik et al. reported the induction of an acute-phase response induced by PDT in a mouse tumor. In the studied mice, there was a systemic upregulation of the investigated indicator, which was the component serum amyloid P. Elevated levels of this indicator in serum and its accumulation in mouse tumors have been described [80]. It has been shown that complement proteins are also involved in the acute-phase response of cancer to PDT, including MBL-A and mouse C-reactive protein [69]. Other tested acute-phase reagents include haptoglobins [86]. PDT induces oxidative stress in the tumor and a state of shock that occurs not only at the site of PDT action but also systemically. In this way, the activation of the acute-phase response is confirmed. These stress signals travel through nerve impulses and are transmitted along the sensory nerve roots to the dorsum of the spinal root and then to the central area of the brain, and finally activate the hypothalamus.

The hypothalamus is the link between the nervous system and the endocrine system through the pituitary gland. The pituitary gland controls the work of the adrenal glands. The paraventricular nucleus of the hypothalamus secretes corticotropin-releasing hormone (CRP). As a further consequence, CRP is delivered to the anterior pituitary via the portal blood vessels of the pituitary gland. The pituitary gland secretes adrenocorticotropic hormone (ACTH). ACTH runs to the adrenal glands and stimulates the production of glucocorticoid hormones. These hormones are rapidly secreted into the systemic circulation. This regulation system is known as the hypothalamic–pituitary–adrenal (HPA) axis. The HPA axis is credited with controlling the stress response and regulating the production of the adrenal glands and other hormones. The HPA axis is the main hallmark of the acute-phase response, dependent on the mobilization of adrenal hormone activity in the context of tumor response to PDT [80].

### 2.2. PDT-Induced Hormonal Activation

**Figure 3:**
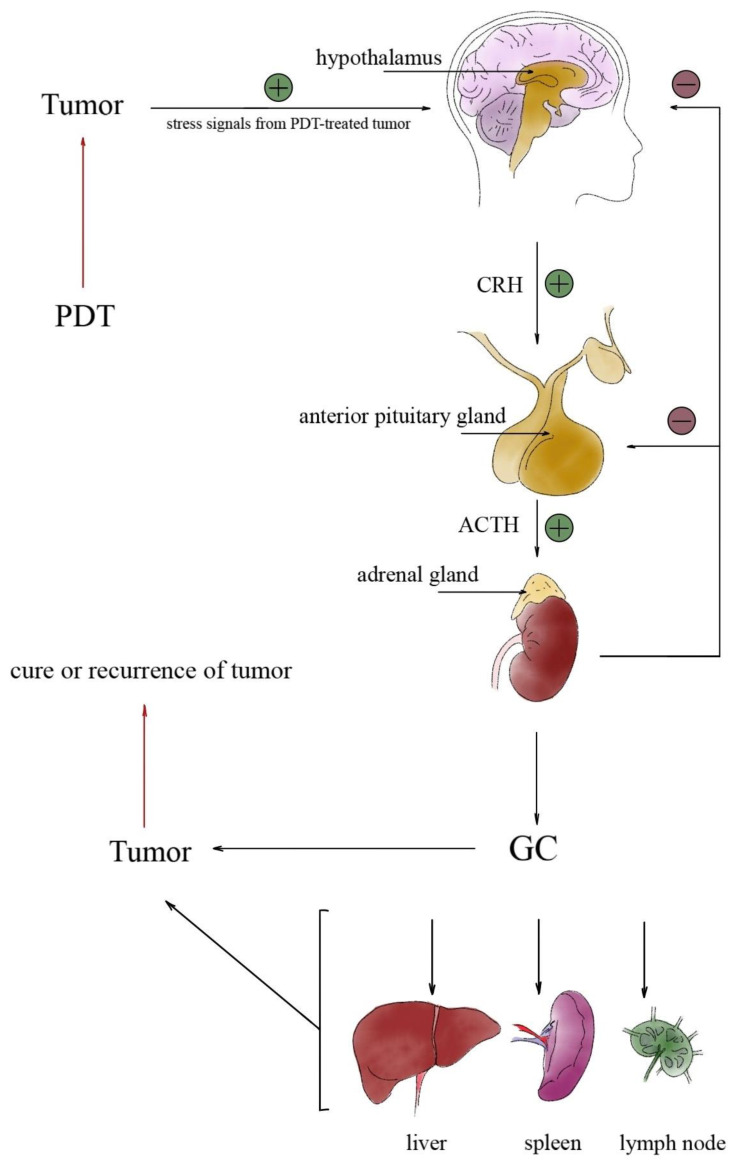
Activation of the hypothalamic–pituitary–adrenal (HPA) axis induced by PDT, resulting in stimulation of the production of adrenal corticosteroid hormones. CRF-corticotropin-releasing hormone; ACTH—adrenocorticotropic hormone; GC—glucocorticoid hormone.

Hormonal activation induced by PDT is the main feature of the acute-phase response [81] and is the basis for mobilization and stimulation of adrenal hormones as a consequence of the use of PDT on cancer cells [85] as shown in Figure 3. All present cellular biomolecules are potentially subject to change by critical oxidation at a level exceeding the cell’s repair capacity [86,87], including cancer cells, on the destruction of which the idea of PDT is based. The oxidative stress generated during PDT leads to the activation of the acute-phase response, which causes a systemic shock to the treated organism. Stress signals are sent as nerve impulses afferently along the sensory roots and the dorsal root of the spinal nerve to the brain. Such an impulse leads to the activation of HPA, starting from the stimulation of the hypothalamus [88], which connects the nervous and endocrine systems through the pituitary gland, whose hormones control the activity of the adrenal glands. The hypothalamus, partly made of neuroendocrine tissue, synthesizes and secretes, among other things, corticotropin-releasing hormone (CRP), which reaches the anterior pituitary through the portal vessels and expresses adrenocorticotropic hormone (ACTH). ACTH released into the blood reaches the adrenal glands, which stimulates the production of GC. The presented HPA system, involving three organs, the hypothalamus, pituitary, and adrenal glands, is responsible for regulating the stress response through a controlled hormonal feedback mechanism [89].

### 2.3. The Role of Glucocorticoids in Tumor Response to PDT

During the acute-phase response, cytokines are released and innate immune cells are activated as well as other inflammatory mediators. These are factors leading to HPA activation [90,91]. The activated HPA axis, through the activity of glucocorticosteroid hormones (GC), modulates the immune response in the body of the treated patient. GCs diffuse freely from circulation across the cell membrane into the cytoplasm by binding to a specific glucocorticoid receptor (GR). Thanks to this, they exert biological activity in various types of cells [92]. GR is a nuclear receptor; it contains the proteins HSP90 and HSP70 and the immunophilin FKBP52. The HSP90 dimer blocks the DNA-binding domain of the GR multi-protein complex. When GC and GR combine, the GR complex dissociates, leaving the DNA-binding subunit free. Subsequently, a homodimer is formed, which is translocated to the cell nucleus. In the promoter regions of target genes, it binds to DNA-responsive elements [92,93]. Glucocorticoids exert their biological activity in a variety of cells through free diffusion (as they are lipophilic) by circulating across the plasma membrane of the cell where they bind to a specific high-affinity glucocorticoid receptor (GR). This receptor is constitutively expressed in every cell type. Upon binding of the glucocorticoid, the GR complex dissociates, leaving the hormone-receptor subunit free [80].

**Figure 4:**
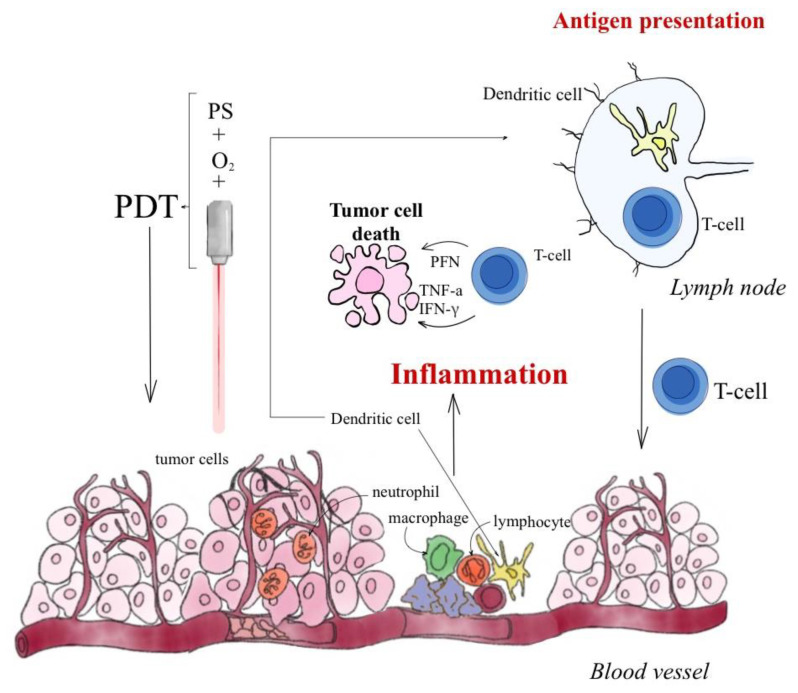
Effect of PDT on the immune response.

The involvement of GC in the tumor response to PDT has been demonstrated by an examination of the levels of neutrophils in mice bearing tumors treated with PDT. In the mice studied, the number of neutrophils increased significantly within 3 h after PDT treatment, but, in adrenalectomized mice, PDT-induced neutrophilia was significantly reduced [94]. This emphasizes the importance of GC in mobilization during inflammation. After PDT, the HSP70 gene is upregulated in the liver and in mice [95], and it is predicted that GCs are involved in this process. The injection of dexamethasone in control mice upregulates HSP70 in the liver, whereas mice injected with IL-6 showed only a slight increase in HSP70 expression. Moreover, in mice with tumors treated with PDT, upregulation of HSP70 in the liver after administration of a glucocorticoid synthesis inhibitor prior to PDT treatment results in downregulation of this gene [91,94]. Figure 4 shows the influence of PDT on the immune response.

In studies in mice with tumors treated with PDT, in which the GR antagonist (mifepristone) was administered 30 min before PDT, a strong induction of the expression of the SAP gene, which is the main mouse reagent in the acute phase, was recorded [94]. The results of the above studies confirm the significant role of adrenal hormones in the expression of PDT-related acute-phase reagents. In addition to indirect effects on cancer, GCs in PDT treatment have a direct effect on tumor cells by regulating the acute-phase response. In Table 2, we outline the role of GC in the treatment of cancer with PDT.

A literature search confirms that PDT is able to elicit a local inflammatory response [97]. Antitumor immunity created during PDT helps with inhibiting tumor growth. However, PDT using PSs such as Photofrin and ALA showed an effect on innate and acquired immunity. To date, these are the two most studied PSs with established protocols that affect immunomodulation. There is still incomplete knowledge about the immunomodulation of other PSs, which are first being tested on animals [98].

PDT mainly damages the membranes and cytoplasm of tumor cells and the vascular system. The cell responds with acute inflammation. Tumor cells secrete immunosuppressive cytokines or other tumor-promoting molecules through non-immunogenic pathways, resulting in an immune-suppressive microenvironment, which inhibits the anticancer immune response.

This acute inflammation is manifested by the secretion of cytokines, leukocyte chemoattractants, growth factors, and other regulators, leading to tissue infiltration by neutrophils, macrophages, mast cells, and NK cells. The PDT inflammatory response can be more pronounced at subcurative doses with a low fluence rate [99]. Detailed studies of immunomodulation have been performed for clinically improved PSs such as Photofrin [100] for obstructing esophageal cancer, obstructing lung cancer, microinvasive endobronchial cancer, gastric cancer, papillary bladder cancer, and cervical dysplasia; Levulan [101] for actinic keratoses; Metvix [102] for actinic keratoses and basal cell carcinoma; Foscan [103] for head and neck cancer; and NPe6 [104] for early lung cancer. Some researchers believe that PDT can stimulate the immune system to work more or less efficiently for a period of treatment time. Further research is needed in the field of PS immunomodulation after PDT treatment.

## 3. Conclusions

PDT leads to the destruction of tumor cells filled with photosensitizer under the influence of light of the appropriate wavelength in the presence of oxygen. This therapy is distinguished by its selectivity towards tumor cells and high safety of use with comparable therapeutic results compared with other methods used in oncology. The action of PDT, in addition to directly inducing the death of cancer cells, leads to activation of the HPA axis. Systemic GCs are an important factor involved in the response to PDT treatment. They participate in therapy and play a main role in the regulation of the total host component in response to tumor PDT, thus protecting the patient from excessive damage to his tissues and controlling the course, rate, and intensity of response to tumor PDT. This is achieved by safeguarding the production of acute-phase reagents in cancer patients treated with PDT by controlling activities related to the destruction of cancer cells, preventing uncontrolled destruction of healthy tissues, promoting removal of killed cancer cells, and supporting healing.

The multi-step mechanisms of GC involvement in the host’s response to tumor PDT appear to contribute positively to therapy. Despite the well-known role of GCs, information on their participation in, and various aspects of their activity in response to, tumor PDT is insufficient to unambiguously determine the action of these hormones and the possibility of clinical use of this knowledge during cancer treatment with PDT. To this end, further commitment to research on the involvement of GCs in tumor PDT is essential. Modulation of GC activity in cancer patients treated with PDT could improve treatment outcomes.

## Figures and Tables

**Table 1:** Categories of cell death after PDT.

Cell Death	Mechanisms	References
paraptosis	Paraptosis occurs independently of caspase activation and is characterized by vacuolation of the cytoplasm, with the formation of vacuoles joined by a single rather than a double membrane and swelling of the mitochondria. In the process of paraptosis, chromatin condensation and cell fragmentation do not occur.	[61]
parthanatos	Parthanatos is the cell’s response to DNA damage and is a caspase-independent process. Cell death is caused by the AIF/MIF complex, which is formed from the AIF factor released from the mitochondria and the MIF macrophage migration inhibitory factor [b].	[62]
mitotic catastrophe	Mitotic catastrophe is a disruption of microtubule organization. The proteins AuroraA, ninein, TOG, and TACC3 enhance aberrant spindle formation and result in apoptotic-like cell death.	[63,64,65]
pyroptosis	Pyroptosis is accompanied by DNA fragmentation and chromatin condensation. Pyroptosis is associated with the activation of one or more caspases.	[66]
necroptosis	Necroptosis can be triggered by multiple stimuli, including surface-associated death receptors, e.g., tumor necrosis factor receptor 1 (TNFR1), DR4/5, and the FAS receptor; by pattern-recognition receptors such as Toll-like receptor 3 (TLR3), TLR4, and Z-DNA binding protein 1 (ZBP1; and by other stimuli that are well described in previous reviews.	[67]
ferroptosis	Ferroptosis is a form of regulated necrotic cell death associated with iron-dependent oxidative modification of phospholipid membranes [h].	[68]

**Table 2:** The putative role of glucocorticosteroid hormones during PDT.

Type	Mechanism
Regulation of the acute-phase response	In the early phase, they promote and downregulate the acute-phase response through a negative feedback loop and regulate it in the late phase after PDT.
Regulation of expression of acute-phase response reagents	They control the generation of acute-phase reagents in the PDT response.
Neutrophil regulation	They participate in the regulation of PDT-induced tumor blood neutrophilia as one of many mediators.
Promoting the progression of induced inflammation	They have a pro-inflammatory effect in the initial stage of Toll-like receptor mRNA stabilization of some proteins, and accelerate its resolution in the advanced stage (as intermediaries in the removal of cells killed by transactivating genes of scavenger receptors, metalloproteinases, IL-10, and TGF-alpha)
Regulation of apoptosis	They have the ability to initiate eosinophils’ apoptosis by regulating their function (preventing their degranulation and releasing cytotoxic proteins) [96].
Influence on tissue remodeling and healing	In the area subjected to PDT, they affect, among other things, the expressions of TGF-alpha, angiogenic factors, fibronectins, growth factors, and other things and promote healing and tissue remodeling.
Alleviating the intensity of the immune response	They increase the expression of genes mediating immunosuppression, which leads to alleviation of the intensity of the anti-cancer adaptive immune response.

## Data Availability

All data have been included.
